# Supplementary material for: Rapid synthesis of phosphor-glass composites in seconds based on particle self-stabilization
Source: Nat Commun. 2024 Feb 3;15:1033. doi: 10.1038/s41467-024-45293-0 (PMC10838289; doi:10.1038/s41467-024-45293-0)
Supplement: Supplementary file 1 — Supplementary Information [file 41467_2024_45293_MOESM1_ESM.pdf]

Supplementary Information

Rapid synthesis of phosphor-glass composites in seconds based on particle self-stabilization

Yongsheng Sun[1], Yuzhen Wang[1], Weibin Chen[2], Qingquan Jiang[2], Dongdan Chen[2], Guoping Dong[2], and Zhiguo Xia[1]*

[1]State Key Laboratory of Luminescent Materials and Devices, Guangdong Provincial Key Laboratory of Fiber Laser Materials and Applied Techniques, Guangdong Engineering Technology Research and Development Centre of Special Optical Fiber Materials and Devices, School of Physics and Optoelectronics, South China University of Technology, Guangzhou 510641, China. *E-mail: xiazg@scut.edu.cn (Z. Xia)

[2]School of Materials Science and Engineering, South China University of Technology, Guangzhou 510641, China.

Corresponding Author

xiazg@scut.edu.cn (Z. Xia)

Table of Contents

Supplementary Fig. 1 The thermal properties characterization of tellurite glass.

Supplementary Fig. 2 The photographs of PGC materials with different YAG:Ce doping concentrations.

Supplementary Fig. 3 Transmission spectra of phosphor-glass composite samples with different YAG:Ce content.

Supplementary Fig. 4 The comparison of YAG:Ce particles before and after embedding tellurite glass.

Supplementary Fig. 5 The prepared YAG:Ce-PGC samples of various shapes and corresponding fluorescence photos.

Supplementary Fig. 6 XRD pattern of YAG:Ce powder, glass, and YAG:Ce-PGC sample.

Supplementary Fig. 7 The Raman spectra of pure YAG:Ce powder, YAG:Ce-PGC fabricated at 650 °C, and tellurite glass.

Supplementary Fig. 8 The TEM characterization of interface between YAG:Ce particles and tellurite glass.

Supplementary Fig. 9 The optical characterization of YAG:Ce-PGC at different melting time.

Supplementary Fig. 10 Fluent simulation.

Supplementary Fig. 11 PL decay curves of YAG:Ce-PGC and YAG:Ce powder.

Supplementary Fig. 12 Comparison of thermal conductivity.

Supplementary Fig. 13 Optical characterization of LuAG:Ce-PGC, YAG:Ce-PGC, and GdAG:Ce-PGC.

Supplementary Fig. 14 Curves of sample temperature versus variation of laser power density.

Supplementary Fig. 15 SEM image of the commercial YAG:Ce-PiG.

Supplementary Fig. 16 CRI and b CCT of the YAG:Ce-PGC samples under different blue laser power densities.

Supplementary Fig. 17 SEM image and corresponding EDS mapping profiles of the YAG:Ce-PGC.

Supplementary Fig. 18 Comparison of thermal stability for YAG:Ce-PGC, YAG:Ce-PiS, and YAG:Ce powder.

Supplementary Table 1 The basic parameter of tellurite glass and YAG:Ce powder.

Supplementary Table 2 I/EQE and absorption of the as synthesized PGC and commercial phosphors.

Supplementary Table 3 EDS analysis of the commercial YAG-PiG.

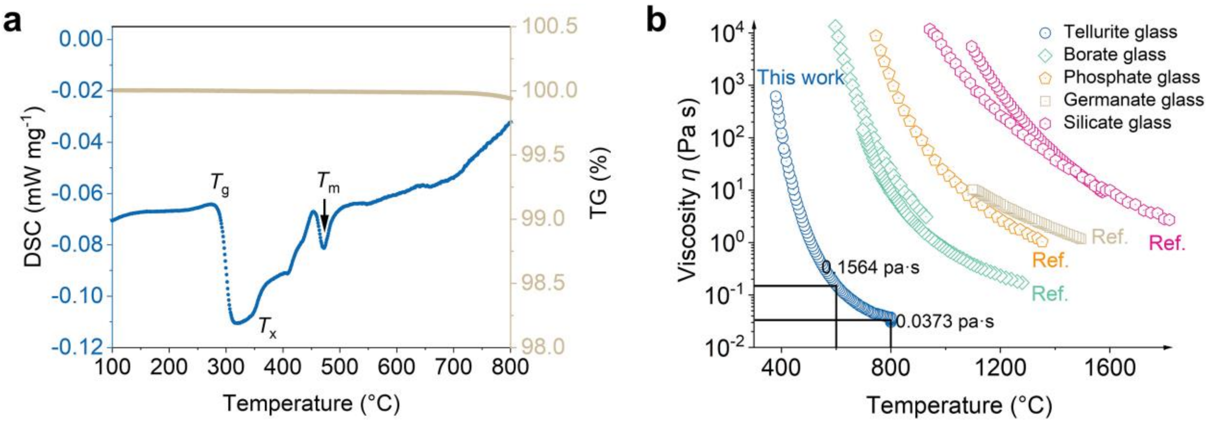

Supplementary Fig. 1 | a DSC-TG curve of the tellurite glass. **b** Viscosity curve of tellurite glass and comparison with other[1, 2, 3]. Orange data line is from Ref. 1, green and red data lines are from Ref. 2, and brown data is from Ref. 3. Source data are provided as a Source Data file.

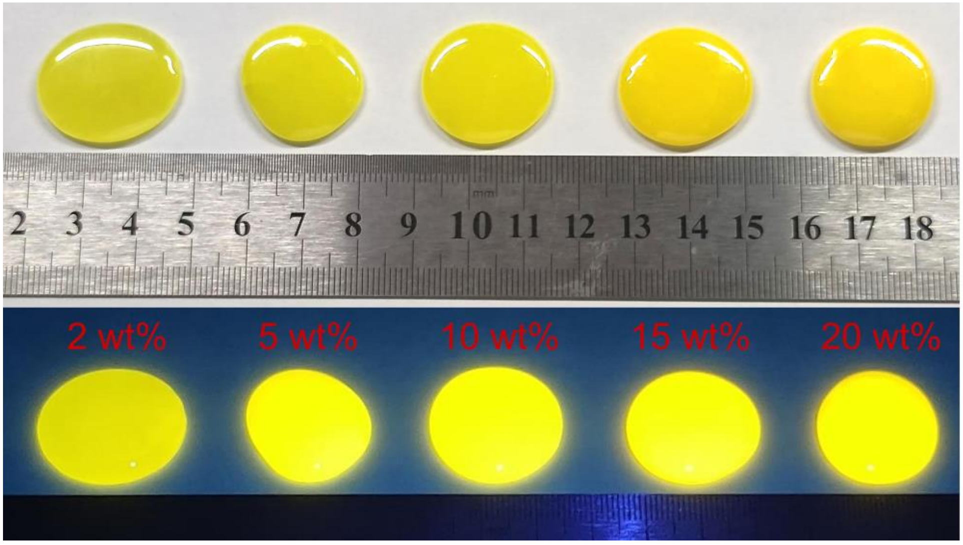

Supplementary Fig. 2 | Photographs of PGC materials with different YAG:Ce doping concentrations and fluorescence photographs at 450 nm excitation.

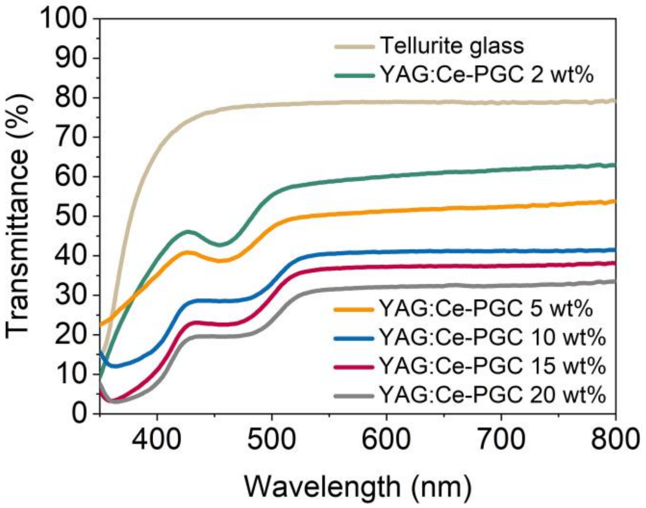

Supplementary Fig. 3 | Transmission spectra of phosphor-glass composite samples with different YAG:Ce content. Source data are provided as a Source Data file.

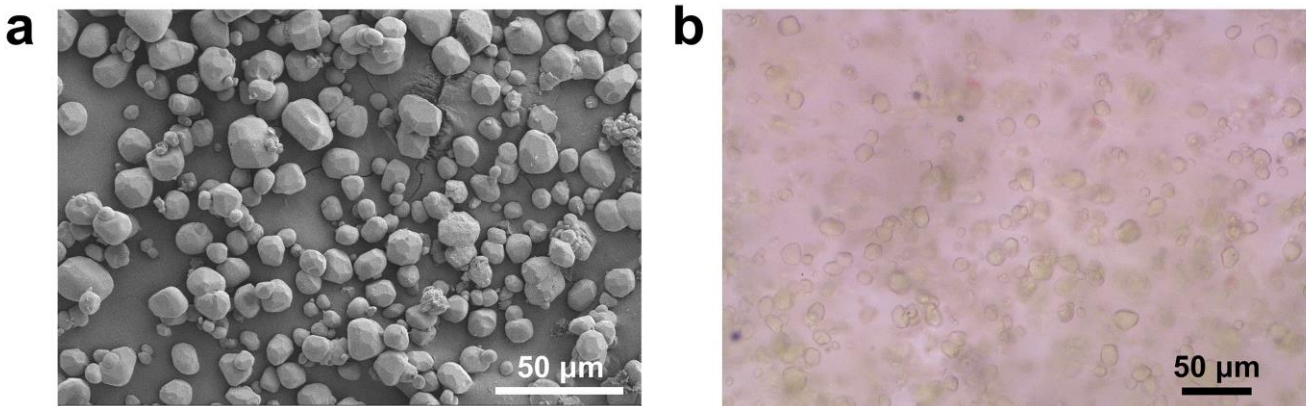

Supplementary Fig. 4 | a Scanning electron microscopy of YAG:Ce phosphor powder. **b** Optical microscope image of the YAG:Ce-PGC sample in transmission mode. Source data are provided as a Source Data file.

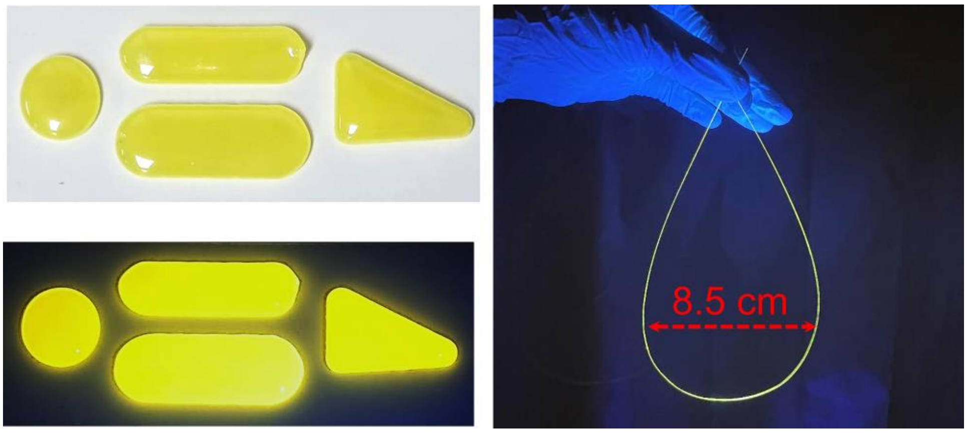

Supplementary Fig. 5 | The prepared YAG:Ce-PGC samples with various shapes and their corresponding fluorescence photos.

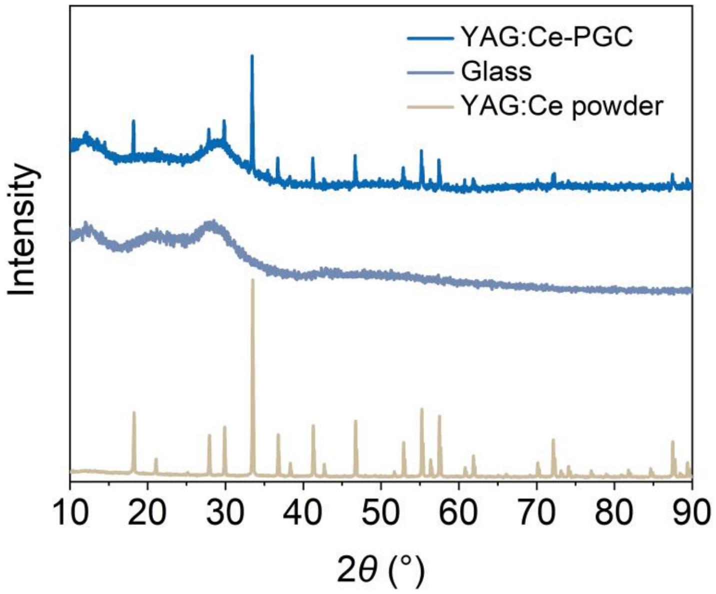

Supplementary Fig. 6 | XRD pattern of YAG:Ce powder, tellurite glass, and YAG:Ce-PGC sample. Source data are provided as a Source Data file.

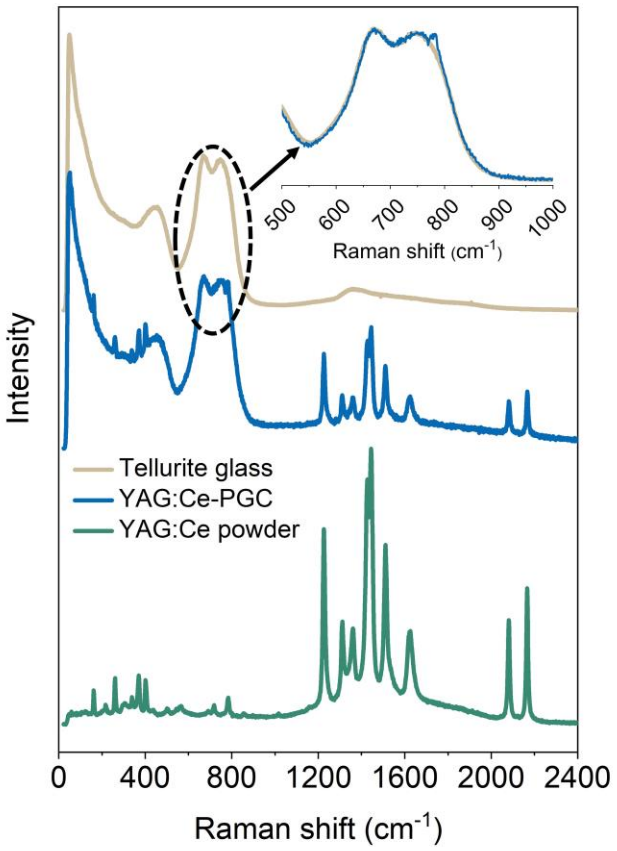

Supplementary Fig. 7 | Raman spectra of pure YAG:Ce powder, YAG:Ce-PGC fabricated at 650 °C, and tellurite glass. The inset is the Raman contrast magnification of tellurite glass and YAG:Ce samples in 500-1000 cm^{-1}. Source data are provided as a Source Data file.

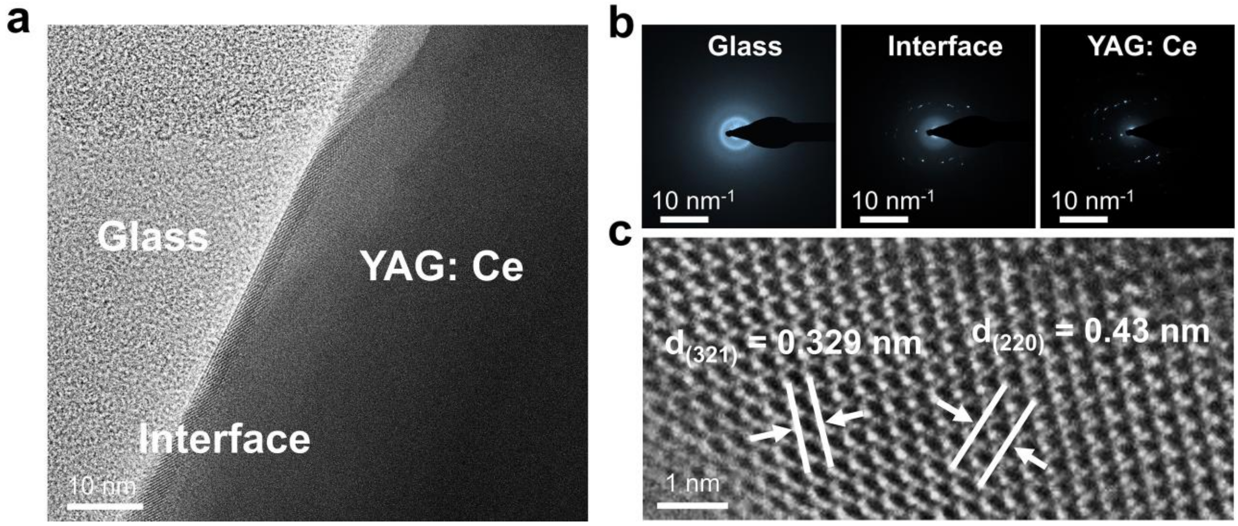

Supplementary Fig. 8 | a TEM image. **b** SAED patterns of YAG:Ce-PGC taken from different locations. **c** HRTEM image of YAG:Ce crystal.

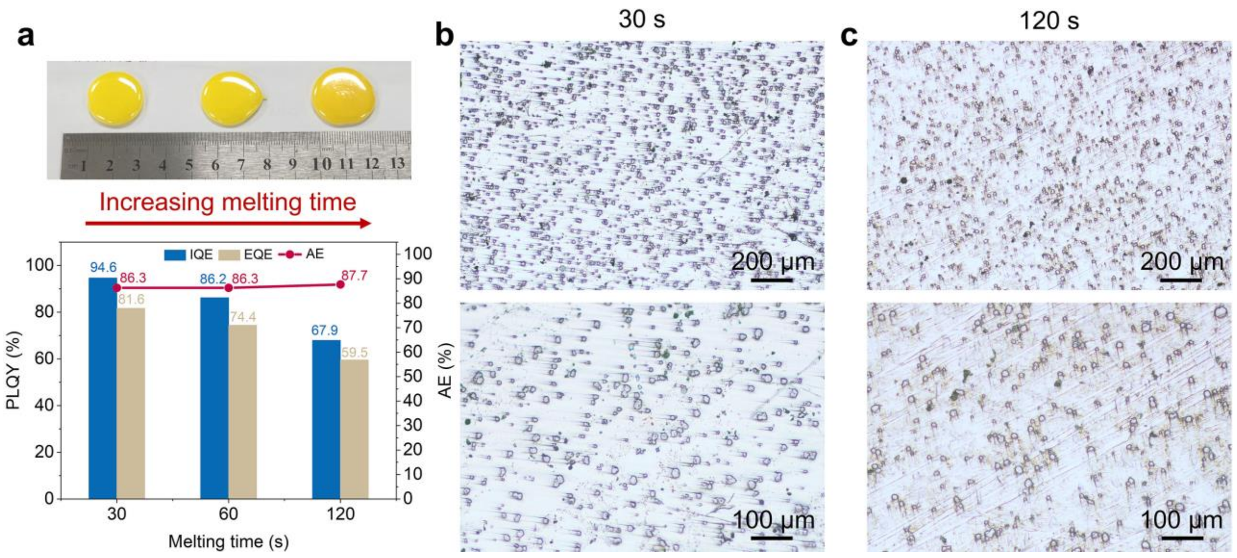

Supplementary Fig. 9 | a Optical photographs and IQE, EQE, and AE of YAG:Ce-PGC with different melting times. Optical microscope image of particle distribution on the surface of YAG:Ce-PGC at melting time **b** 30 s and **c** 120 s. Source data are provided as a Source Data file.

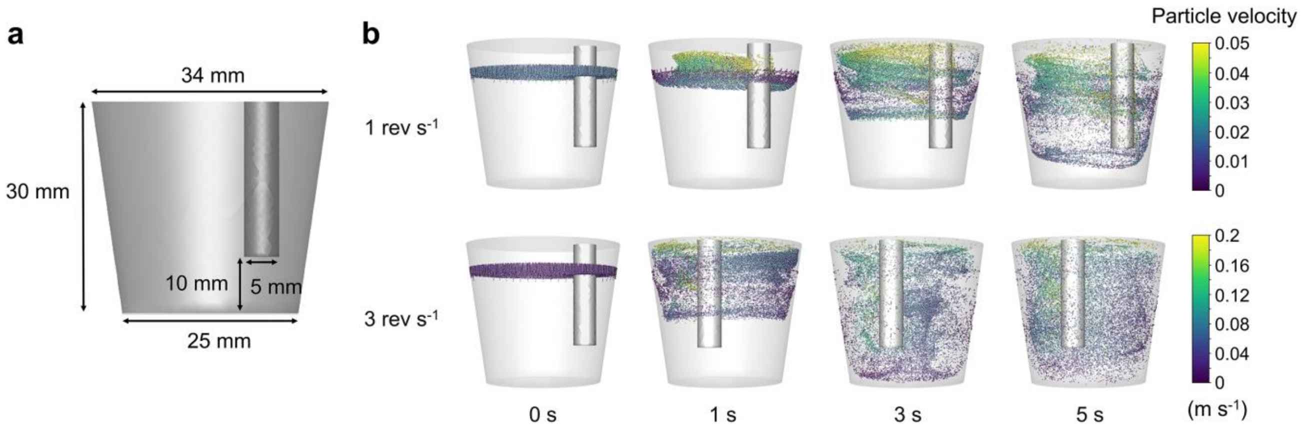

Supplementary Fig. 10 | a Geometry model. Images used courtesy of ANSYS, Inc. **b** The time-dependent fluent simulation dispersion of YAG particles in tellurite glass melt (at different stages of 0, 1, 3 and 5 s) under 1 rev s^{-1} and 3 rev s^{-1} stirring speeds, and the depth of the particle color represents the velocity of the particle at this moment. Images used courtesy of ANSYS, Inc.

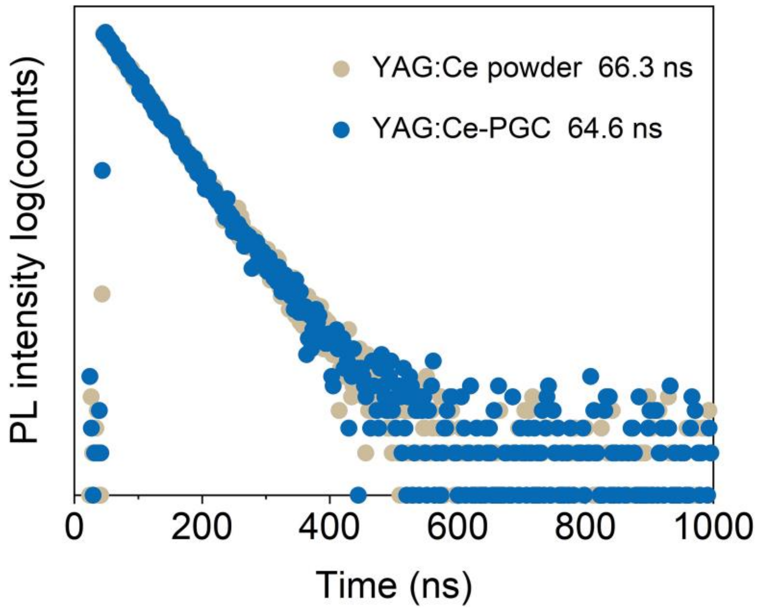

Supplementary Fig. 11 | PL decay curves of YAG:Ce-PGC and YAG:Ce powder. Source data are provided as a Source Data file.

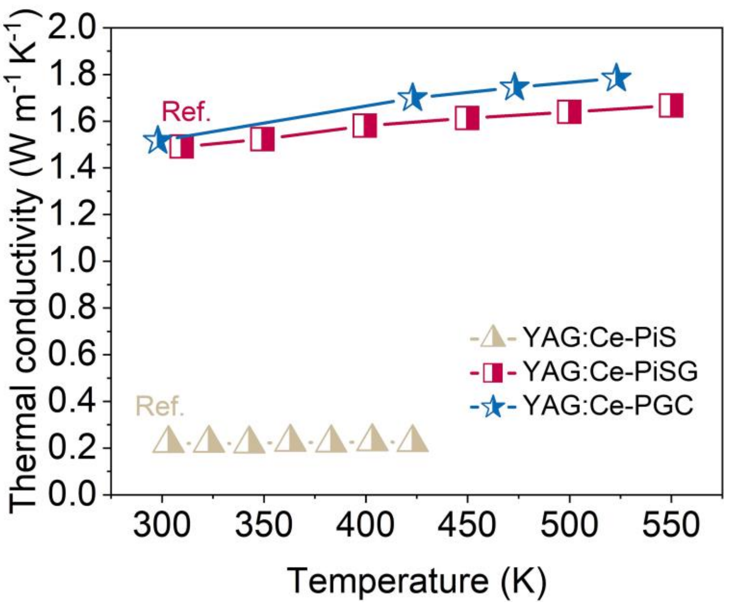

Supplementary Fig. 12 | Thermal conductivity of 20 wt% YAG:Ce-PGC, YAG:Ce phosphor in silica glass (YAG:Ce-PiSG, YAG:Ce-PiSG is prepared by spark plasma sintering technology with sol-gel derived silica glass as matrix material), and YAG:Ce phosphor in silicone (YAG:Ce-PiS)[4], brown and red data lines are from Ref. 4. Source data are provided as a Source Data file.

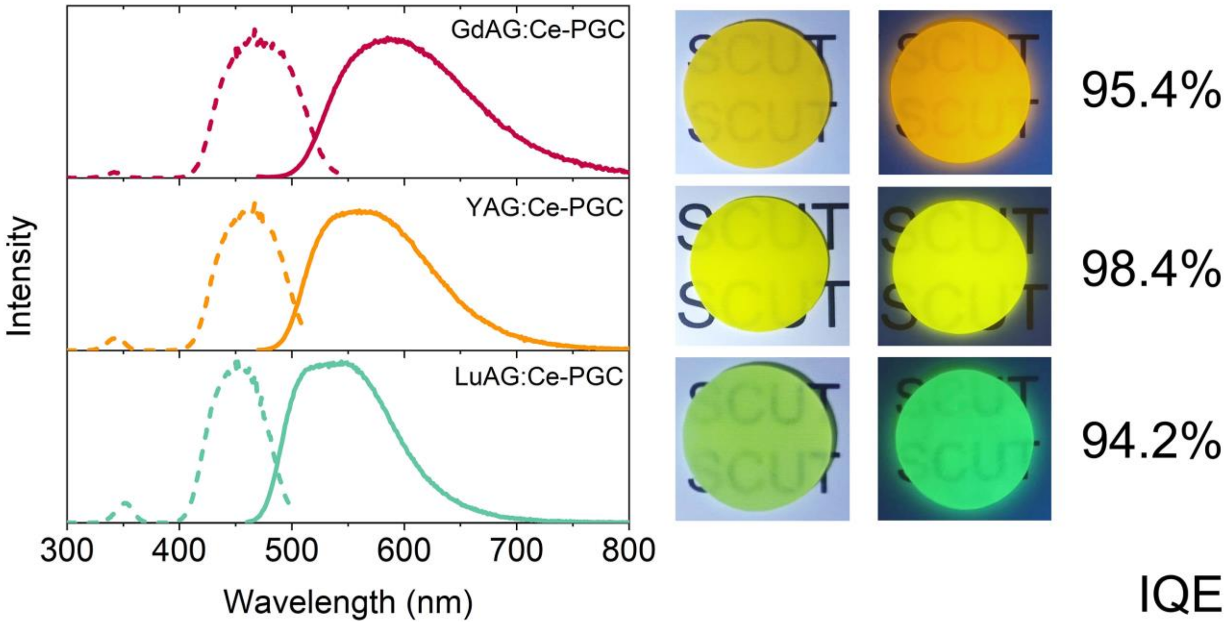

Supplementary Fig. 13 | PL and PLE spectra of LuAG:Ce-PGC, YAG:Ce-PGC, and GdAG:Ce-PGC, and optical and fluorescence photographs at 450 nm excitation. Source data are provided as a Source Data file.

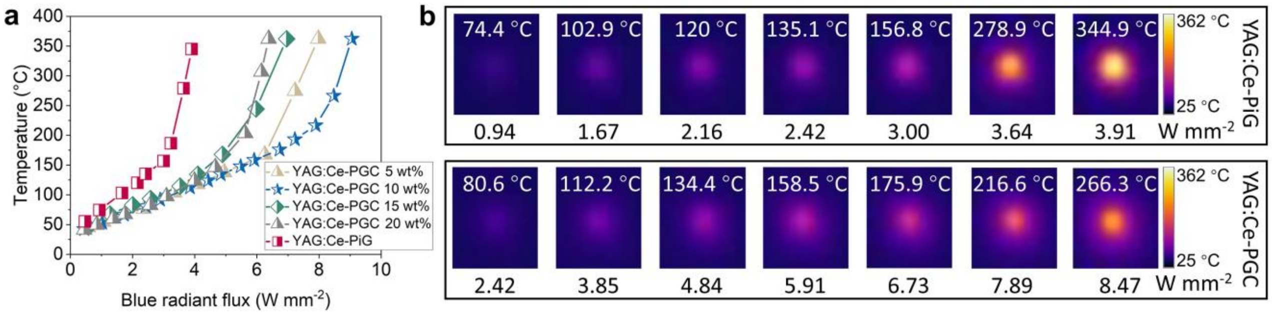

Supplementary Fig. 14 | a Curves of sample temperature versus variation of laser power density. **b** Thermal infrared images of the commercial YAG:Ce-PiG and the YAG:Ce-PGC 10 wt% samples at changed laser power density. Source data are provided as a Source Data file.

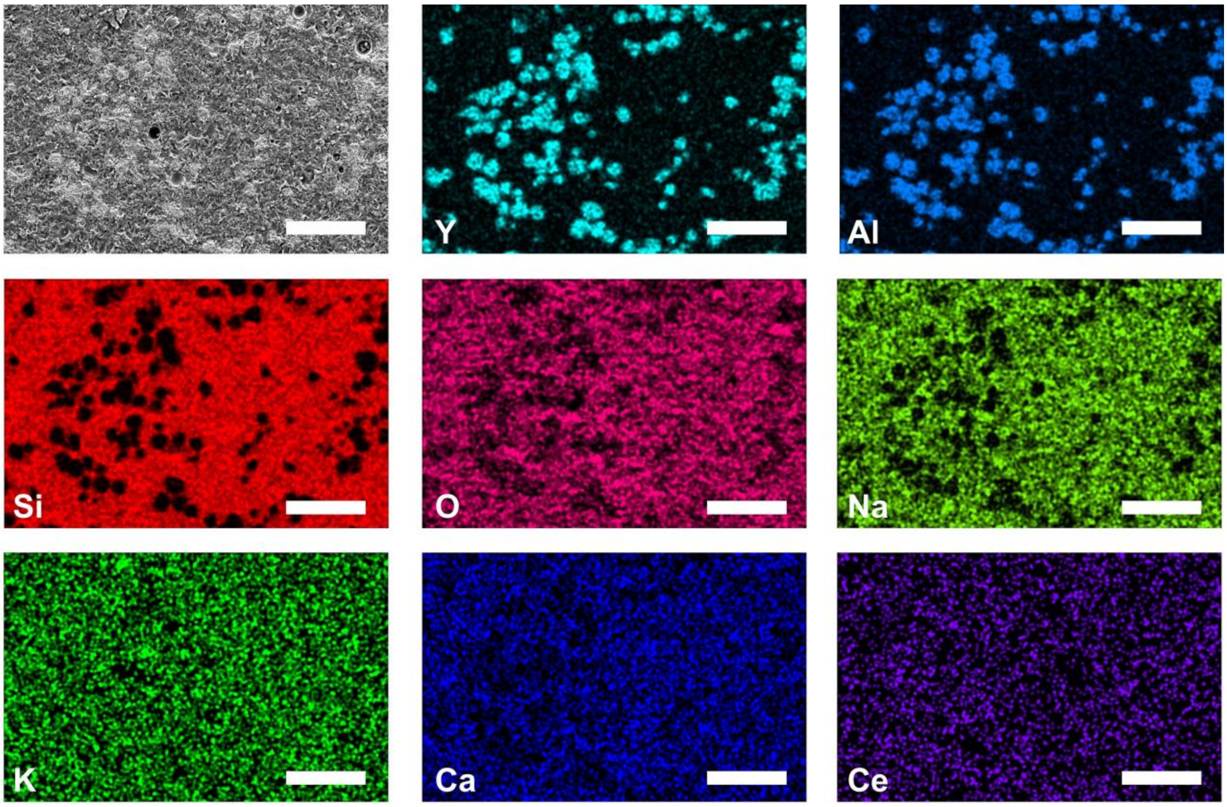

Supplementary Fig. 15 | SEM image and corresponding EDS mapping profiles of the commercial YAG:Ce-PiG (scale bar, 80 μm).

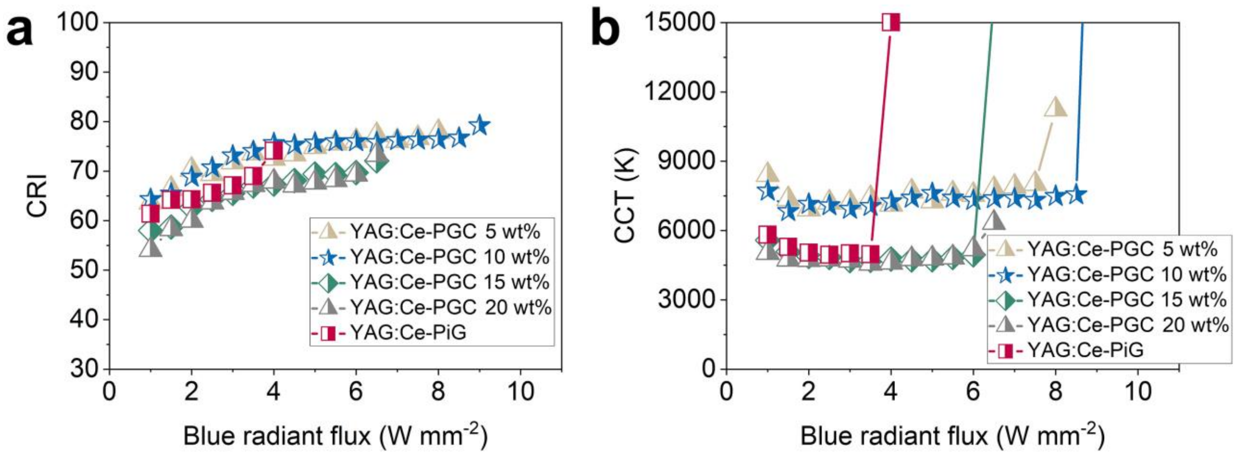

Supplementary Fig. 16 | a CRI and **b** CCT of YAG:Ce-PGC samples under different blue laser power densities. Source data are provided as a Source Data file.

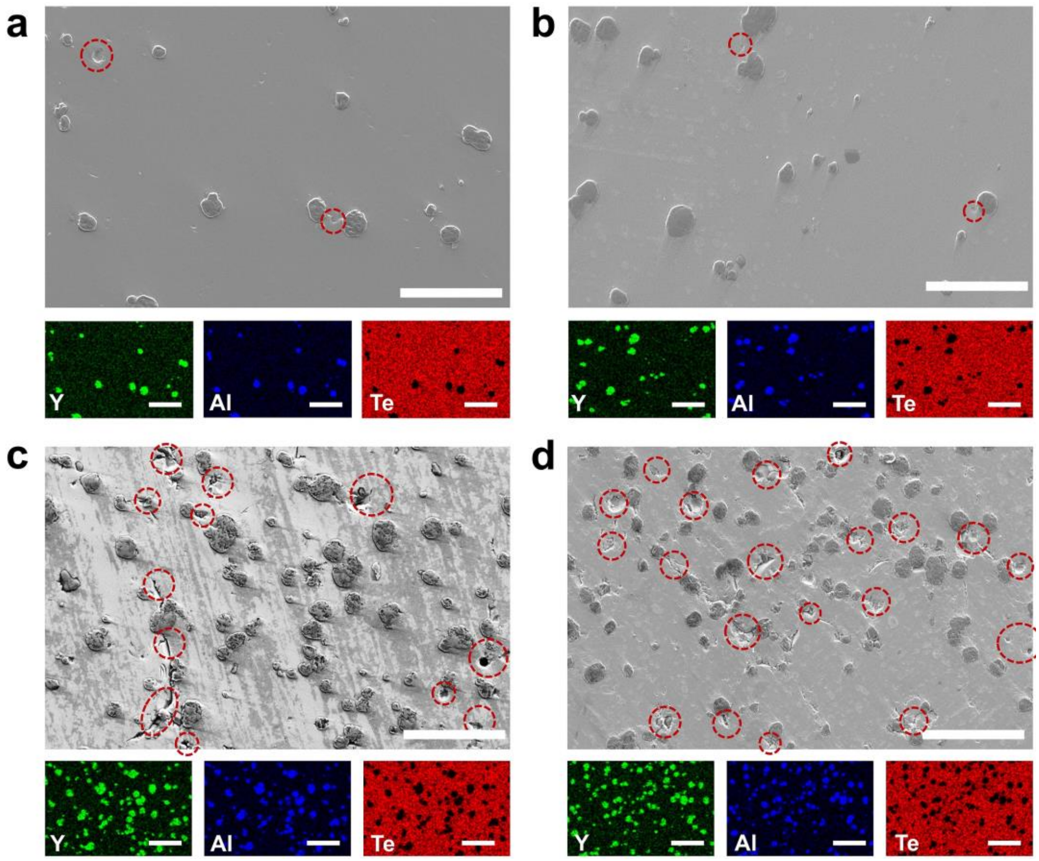

Supplementary Fig. 17 | SEM image and corresponding EDS mapping profiles of the YAG:Ce-PGC with **a** 5 wt%, **b** 10 wt%, **c** 15 wt%, and **d** 20 wt% YAG:Ce phosphor (scale bar, 80 μm).

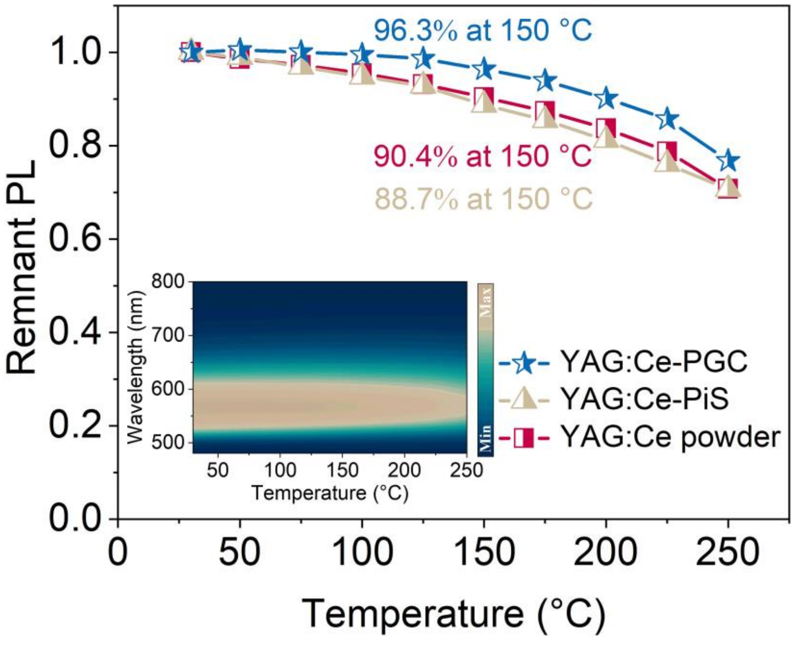

Supplementary Fig. 18 | Integrated PL intensities of YAG:Ce-PGC, YAG:Ce-PiS, and YAG:Ce powder as a function of the temperature, the inset is contour plot of the PL spectra (*y*-axis) and temperature (*x*-axis) exhibiting the thermal stability of YAG:Ce-PGC. Source data are provided as a Source Data file.

Supplementary Table 1. The basic parameter of tellurite glass and YAG:Ce powder.

Tellurite glass	Dielectric constant $\boldsymbol{\varepsilon}_{\mathbf{G}}$	26.5196
	Density $\boldsymbol{\rho}$	5.02 g cm^{-3}
	Refractive index $\boldsymbol{n}_{\mathbf{G}}$	1.97
	Surface energy of glass melt $\boldsymbol{\sigma}_{\mathbf{G}}$	0.141 J m^{-2}
YAG:Ce powder	Dielectric constant $\boldsymbol{\varepsilon}_{\mathbf{Y}}$	12.5952
	Particle radii $\boldsymbol{R}$	5.4 µm
	Density $\boldsymbol{\rho}$	4.70 g cm^{-3}
	Refractive index $\boldsymbol{n}_{\mathbf{Y}}$	1.84
Parameter	Planck constant **h**	6.626×10^{-34} J s
	Boltzmann constant $\mathbf{k_B}$	1.38×10^{-23} J K^{-1}
	Contact angle between Tellurite glass and YAG ceramic $\boldsymbol{\theta}$	43.5°
	Melting temperature $\boldsymbol{T}$	923 K

Supplementary Table 2. I/EQE and AE of the as synthesized PGC and commercial phosphors.

Samples	Excitation wavelength (nm)	IQE (%)	EQE (%)	AE (%)
YAG: Ce powder	450	99.4	74	74.5
2 wt% YAG:Ce-PGC	450	92.3	78.7	85.3
5 wt% YAG:Ce-PGC	450	95.5	82.4	86.3
10 wt% YAG:Ce-PGC	450	98.4	85.4	86.8
15 wt% YAG:Ce-PGC	450	98	85	86.7
20 wt% YAG:Ce-PGC	450	97.9	85.1	86.9
LuAG:Ce powder	450	99	59.7	60.3
5 wt% LuAG:Ce-PGC	450	94.2	78.5	83.3
GdAG:Ce powder	450	98.4	59.5	60.5
5 wt% GdAG:Ce-PGC	450	95.4	83.6	87.6

Supplementary Table 3. EDS analysis of the commercial YAG-PiG.

Element	wt%	at%
Y	14.42	3.79
Al	5.33	4.61
Si	29.7	24.7
Na	7.26	7.38
K	1.51	0.9
Ca	2.2	1.28
Ce	0.34	0.06
O	39.23	57.28

Supplementary references

1. Ehrt, D., Flügel, S. Electrical conductivity and viscosity of phosphate glasses and melts. *J. Non-Cryst. Solids* **498**, 461-469 (2018).

2. Ehrt, D. K., Ralf. Electrical conductivity and viscosity of borosilicate glasses and melts. *Phys. Chem. of Glasses-B* **50**, 165-171 (2009).

3. Riebling, E. F. Structure of Molten Oxides. I. Viscosity of GeO_2, and Binary Germanates Containing Li_2O, Na_2O, K_2O, and Rb_2O. *J. Chem. Phys.* **39**, 1889-1895 (1963).

4. Zhou, B., *et al.* Enhancing the performance of Ce:YAG phosphor-in-silica-glass by controlling interface reaction. *Acta Mater.* **130**, 289-296 (2017).
